# Immune regulation in gastric adenocarcinoma is linked with therapeutic efficacy and improved recovery

**DOI:** 10.3389/fgene.2023.1238248

**Published:** 2023-08-11

**Authors:** Zhenglei Xu, Ximin Lin, Haotian Zeng, Xiaoxin Ma, Ghulam Nabi, Zain Ul Abidin, Luolin Wang, Lisheng Wang

**Affiliations:** ^1^ Department of Gastroenterology, Shenzhen People’s Hospital, The Second Clinical Medical College, The First Affiliated Hospital, Jinan University, Southern University of Science and Technology, Shenzhen, Guangdong, China; ^2^ The Second Clinical Medical College, Jinan University, Shenzhen, Guangdong, China; ^3^ Institute of Nature Conservation, Polish Academy of Sciences, Krakow, Poland; ^4^ Department of Intensive Care Unit, Kabir Medical College, Peshawar, Pakistan

**Keywords:** adenocarcinomas, diagnosis, gastric cancer, biomarker, therapy

## Abstract

Adenocarcinomas are one of the most common histological types of gastric cancer. It has been ranked fifth among common cancers and is the third among death causing cancers worldwide. The high mortality rate among patients with gastric cancer is because of its silent evolution, genetic heterogeneity, high resistance to chemotherapy as well as unavailability of highly effective therapeutic strategy. Until now a number of several treatment strategies have been developed and are being practiced such as surgery, chemotherapy, radio therapy, and immunotherapy, however, further developments are required to improve the treatment responses and reduce the side effects. Therefore, novel personal therapeutic strategies based on immunological responses should be developed by targeting different check points and key immune players. Targeting macrophages and related molecular elements can be useful to achieve these goals. In this minireview, we discuss the available treatment options, molecular underpinnings and immunological regulations associated with gastric adenocarcinoma. We further describe the possible check points and immunological targets that can be used to develop novel therapeutic options.

## Introduction

Gastrointestinal cancers of stomach, esophagus, colon, pancreas and liver are considered as the leading cause of cancer morbidity and mortality (Moehler et al., 2022). These cancers have caused 3.2 million deaths in 2020 due to lack of convenient and effective therapeutic options for patients with severe symptoms (León-Gonzalez et al., 2014; Ahmed and Othman, 2020; Moehler et al., 2022). Gastric adenocarcinoma is considered the 5th most common cancer type and ranked 3rd among high deaths causing cancers worldwide (Hudler, 2012; Moehler et al., 2022). It is most common in Asian populations, and prognosis and effectiveness of treatments depends on regional differences in disease progression and etiology (Ivey et al., 2022). The molecular and epidemiologic background of gastric cancers is unique. For example, intestinal gastric adenocarcinoma develops from chronic gastritis caused by *Helicobacter pylori*, resulting in intestinal metaplasia, atrophy, and intraepithelial neoplasia (dysplasia), ultimately leading to cancer (Yakirevich and Resnick, 2013).

**FIGURE 1:**
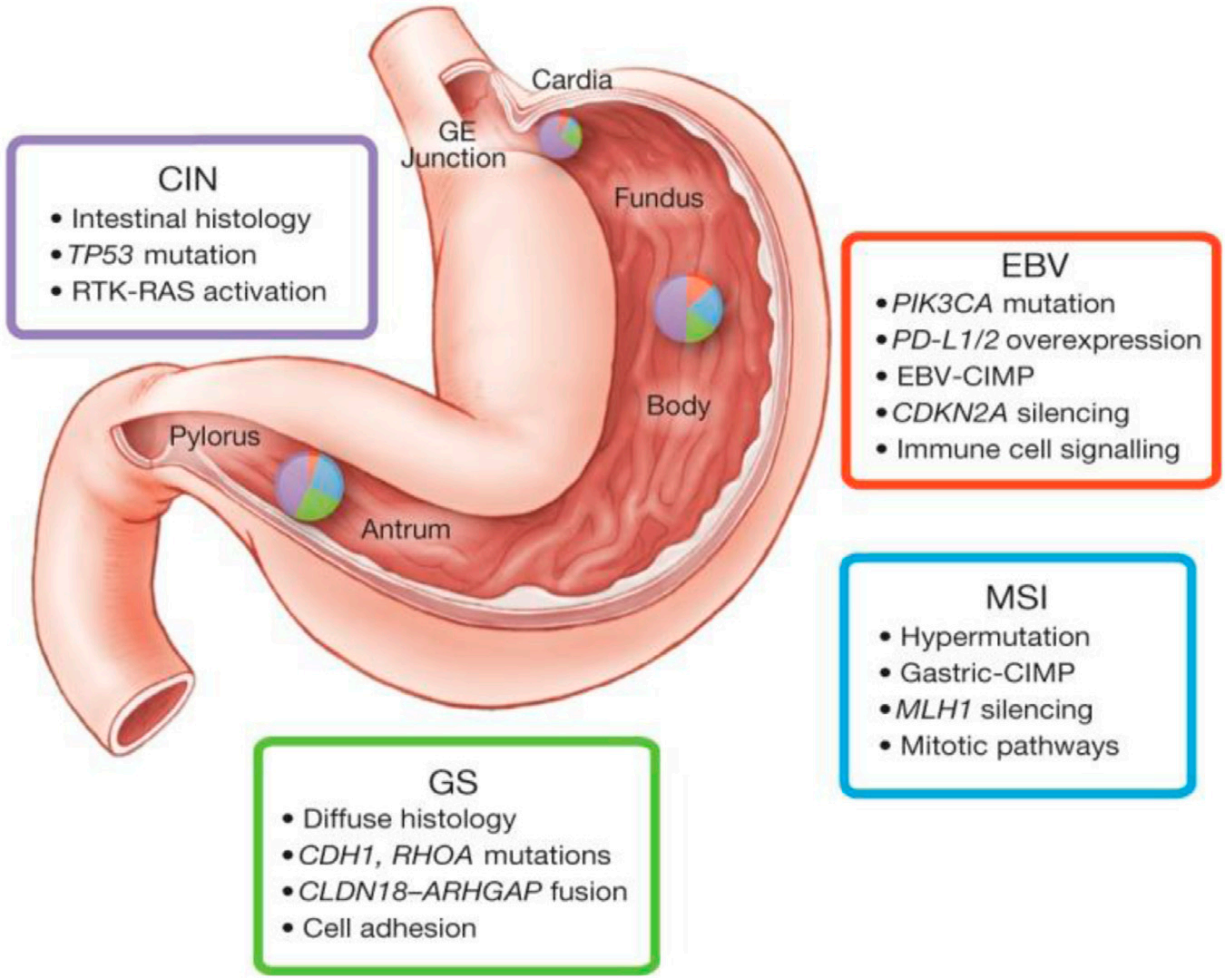
This figure depicts some key features of gastric cancer subtypes based on molecular biology. Inset charts represent the distribution of molecular subtypes in gastric tumours obtained stomach. The molecular classification of gastric cancer indicates four prominent genomic subtypes (Genomically stable tumours, EBV-infected tumours; chromosomally unstable tumours, and Microsatellite instability (MSI) tumours. This figure was adopted from The Cancer Genome Atlas Research Network according to the rights and permissions statement of the publisher (Comprehensive molecular characterization of gastric adenocarcinoma., 2014). (CIN, chromosomal instability; EBV, Epstein-Barr virus; MSI, Microsatellite instability; GS, genomically stable).

The available treatments include chemotherapy, radiation and surgery, where radiation therapy can be applied before or after surgery. Moreover, radiation therapy is also used in combination with chemotherapy for enhanced sensitization of tumor (Phan et al., 2023). Gastrointestinal cancers treatment through immunotherapeutic options include vaccine therapies (dendritic cell-based vaccines, peptide, protein, whole tumor cells), adoptive T cell transfer cytokines (IL-10, GM-CSF interferon-γ, interleukin-2), and checkpoint inhibitors (CTLA-4, PD-1, PD-L1) (Phan et al., 2023). However, the most common treatment strategy for gastric adenocarcinoma is surgical resection (He et al., 2021; Phan et al., 2023). Even though adjuvant therapies have improved treatment responses, patients can still develop metastases and become resistant to treatment (Phan et al., 2023). Therefore, further work is needed to develop more effective therapeutic options and identify novel drug targets to improve treatment and survival (Figure 1).

Esophagogastric adenocarcinomas can be successfully treated with immunotherapy alone or in combination with chemotherapy, whether the tumor is epidermal growth factor receptor 2 (Her2) positive or negative (Moehler et al., 2022). Chemotherapy is typically recommended to reduce symptoms, control disease and prolong survival in patients with surgically unresectable gastric cancers. On the other hand, patients with tolerance to high toxicity are treated with fluoropyrimidine and platinum double with an additional chemotherapy (Cassetta and Pollard, 2018; Ivey et al., 2022). Despite the availability of these treatment options, the selection and efficacy of the treatment is challenging due to intra-tumoral heterogeneity mediated treatment resistance (Ivey et al., 2022).

## Prominent treatment strategies in practice

Due to the lack of robust prognostic data for gastric adenocarcinoma, it is crucial to determine the stage using the current tumour-node-metastasis (TNM) model that incorporates biomarkers (Trout et al., 2022). To guide T and N staging, endoscopic ultrasonography (EUS) with or without fine-needle aspiration is mainly used. In addition to Computed Tomography (CT) or positron emission tomography (PET) imaging, (Spolverato et al., 2015), staging laparoscopy is the gold standard for diagnosing gastric adenocarcinoma. Further investigations are required to confirm whether polarized enhanced laparoscopy and 3D high-definition laparoscopy offer additional advantages over 2D laparoscopy (Trout et al., 2022).

### Perioperative therapy

Multimodality approaches have been reported with better treatment outcomes in the case of gastric adenocarcinoma. In patients with localized adenocarcinoma, perioperative chemotherapy (three preoperative and three postoperative cycles of epirubicin plus cisplatin and 5-fluorouracil) plus surgery was reported to be a more favorable approach than surgery alone (Cunningham et al., 2006). As a standard-of-care approach, 5-fluorouracil plus cisplatin for two or 3 months prior to surgery and four or 5 months after surgery was determined to be an appropriate perioperative chemotherapy regimen (Cunningham et al., 2006; Slagter et al., 2018; Janjigian et al., 2021).

### Chemoradiotherapy

In patients with resectable gastric adenocarcinoma, postoperative chemotherapy has a limited role (Slagter et al., 2018). There is an interest in combining radiotherapy with chemotherapy in the neoadjuvant cancer treatment (Slagter et al., 2018).

### Endoscopic resection

Adenocarcinoma of the stomach in the early stages can be resected using endoscopic submucosal dissection (ESD), a technique pioneered in Japan (Ahmed and Othman, 2020). In most cases of superficial gastric adenocarcinoma, ESD is recommended as the treatment of choice. For larger tumours, including those with ulcerations, ESD is more effective than endoscopic mucosal resection (EMR), however, ESD also requires greater skill than EMR (Ahmed and Othman, 2020).

### Surgery

A gastrectomy with circumferential and linear tumour-free margins is the main principle of the surgery. Tumor stage, histological subtype, and location determine how much surgical resection is possible (Spolverato et al., 2015; Moehler et al., 2022). Typically, proximal margins should be 5 cm, but they can be increased to 8 cm for diffuse type tumors. In comparison with a D2 dissection, surgeons prefer a D1 dissection because the risk of morbidities is perceived to be higher, and the survival outcomes are minimally improved. Studies have shown that higher mortality and morbidities are observed in the D2 group gastrectomy, because of the surgeon being naïve to pancreatic and splenic resections (Amin et al., 2017). Additionally, bursectomy has been shown to reduce the risk of peritoneal metastases by en-bloc resection of the post-gastric cavity lining, which might contain free cancer cells and/or micro-metastases. It can be used as an alternative surgical procedure, however, it does not provide a long-term post-surgery advantage (Amin et al., 2017; Janjigian et al., 2021).

### Minimally invasive surgery

The laparoscopic approach to gastrectomy is the most popular minimally invasive method, which offers similar survival rates to open gastrectomy when performed by experienced surgeons. A laparoscopic approach has been shown to improve postoperative recovery times, decrease blood loss, shorten hospital stays, and improve general health. Still, a large level improvements are required for laparoscopic gastrectomy to replace surgical approaches (He et al., 2018). The use of robotics in gastrectomy has evolved as an essential component of surgical armamentarium, and its safety and efficacy have been well documented. The robot-assisted gastrectomy uses a high-definition 3D camera, which ensures stable and magnified views, as well as tremor reduction (He et al., 2018).

### Endoscopic resection

In early-stage tumours, endoscopic resection can be a viable alternative to open surgery. A larger tumour can be removed with either an EMR or an ESD, which requires a higher level of surgical skill. It is important to consider the depth, diameter, histological grade, and ulcerative component of the tumour when determining whether endoscopic resection is feasible (Hirata et al., 2023).

### Molecular and immunological interpretations in gastric adenocarcinoma

**FIGURE 2:**
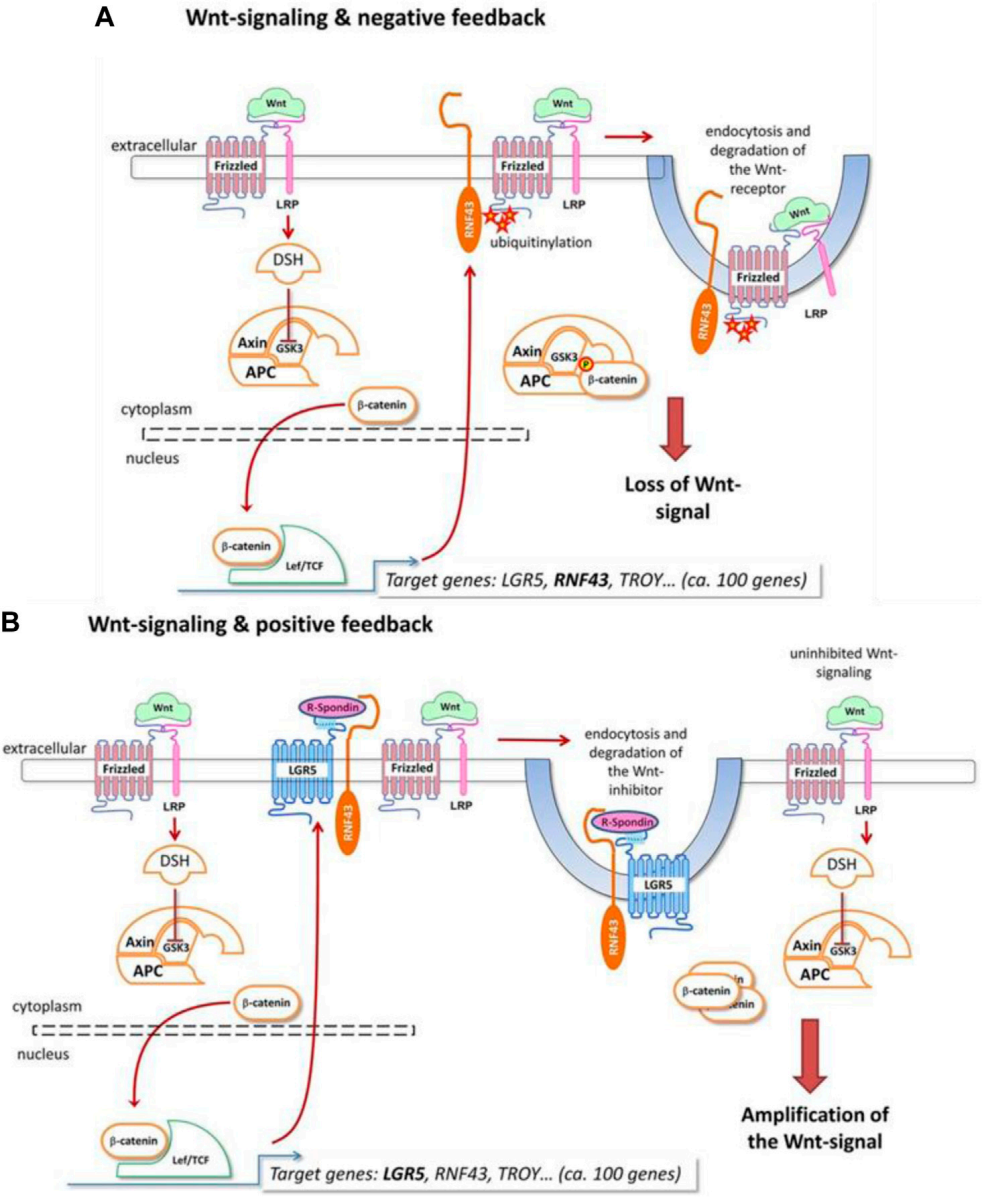
This figure shows Want signaling pathway, and the associated regulatory mechanisms. Negative or positive feedback mechanisms associated with Wnt signaling pathway depend on the interaction between different molecules, which determine the function and importance of this pathway in gastric adenocarcinoma. In this pathway, Wnt ligand is facilitated to bind to FZD-LRP5/6 complex, while the destruction complex is inhibited by dishevelled (DVL), which prevents the degradation of β-catenin. β-catenin is accumulated in the cytoplasm followed by translocation into the nucleus, thereby activating transcription factors (Figure 2). The negative regulators of the Wnt signaling pathway are RNF43 and LRP1B **(A)**. In case of mutation of loss of function of RNF43 or LRP1B, Wnt signaling is increased **(B)**. The figure was adopted from the article published by Holm et al. (Holm et al., 2023) following the rights and permissions statement from the licensing agency/publisher. (DC; destruction complex, RNF; Ring Finger Protein, LRP; lipoprotein receptor-related protein, TCF; T-cell factor, LEF; lymphoid enhancer factor, LGR; Leucine-rich repeat-containing G-protein-coupled receptor, APC/MCC; Familial Adenomatous Polyposis).

Unveiling the underlying molecular mechanisms is crucial to elucidate immune evasion, therapeutic efficacy, and progression/development of cancer (Moehler et al., 2022). Therefore, previous studies have largely reported the molecular profiling of gastric adenocarcinoma, which have indicated deregulation of distinct oncogenic pathways such as proliferation, Wnt–β-catenin (Figure 2), and NF-κB (Cristescu et al., 2015). In the case of primary gastric adenocarcinomas, the most comprehensive molecular characterization was provided by “The Cancer Genome Atlas (TCGA)” in 2014 (Cancer Genome Atlas Research Network, 2014). Based on integrative clustering analysis of data received from six molecular platforms “DNA methylation, somatic mutation profiles and copy number variations, microRNA sequencing, proteomics analyses, and gene expression,” the primary gastric adenocarcinomas were classified into four subtypes (Chromosomal instability, EBV-positive, GI, MSI) (Comprehensive molecular characterization of gastric adenocarcinoma., 2014). In another molecular profiling study, published by the Asian Cancer Research Group (ACRG), 300 primary gastric adenocarcinomas were classified into four subtypes (MSI subtype, mesenchymal-like subtype, *TP53*-inactive subtype and *TP53*-active subtype) (Cristescu et al., 2015). Previous studies based on meta-analysis of bulk expression data related to gastric adenocarcinomas have indicated that high proportions of CD8^+^ T cells and M1 macrophages were associated with a more favorable prognosis of intestinal gastric cancer (Zeng et al., 2019).

### Molecular targets

Gastric adenocarcinomas possess a number of unique molecular features that can be targeted clinically. *KRAS* alterations occur in several gastric adenocarcinomas, where *KRAS*G13D and *KRAS*G12D are common mutations, which can be targeted. Pan-KRAS agents can be used to target *KRAS* amplifications mediated overexpression. Furthermore, overexpression of HER2 encoding gene *ERBB2* can be targeted along with targeting mutations associated with deficiencies in DNA damage repair (Hirata et al., 2023). Earlier therapeutic attempts involving anti-EGFR or anti-VEGF antibodies were not effective, therefore, next-generation therapies based including, vaccines, antibody-drug conjugates, antibodies, and cell therapies can be used to treat gastric adenocarcinoma. In case of gastric adenocarcinoma, the frequently altering oncogene *KRAS* has been reported to be involved in development of tumor (Canon et al., 2019; Hirata et al., 2023), thus activated KRAS can be targeted in aforementioned cancer using the combination of SHP2 and MEK inhibitors. Overexpression of *ERBB2* (encoding HER2) depends on stage of disease stage, subtype of tumour other relevant variables (Chung et al., 2021). HER2 can be targeted by FDA approved trastuzumab, monoclonal antibody in combination with chemotherapy in patients with advanced-stage HER2-positive gastric adenocarcinoma (Safran et al., 2022; Hirata et al., 2023). Several HER2-targeting therapeutic options in combination with immune checkpoint inhibitors have shown promising results such as, trastuzumab deruxtecan; antibody–drug conjugate consisting of a humanized, monoclonal, anti- HER2 antibody bound to a cytotoxic topoisomerase I inhibitor (Wheeler et al., 2021). Several potential inhibitors for alterations in expression of receptor tyrosine kinases and RAS–RAF or PI3K–AKT–mTOR signalling cascades are active in gastric adenocarcinoma. Genomic alterations in *mTOR PI3KCA*, and *PTEN* have been reported in gastric adenocarcinoma, such that *PI3KCA* and *mTOR* mutations are potential activating alterations, while PTEN (loss-of-function mutation) activates PI3K–mTOR signalling, which could be targeted using specific inhibitors (Mishra et al., 2021).

### Targeting stem cell signalling pathways

Immune regulation is important in cancer progression and treatment (Zeng et al., 2019; Varadé et al., 2020). In this regard transcriptional coactivators (such as YAP1) of the highly conserved developmental pathway known as HIPPO signalling pathway are responsible for immunosuppression. In gastric adenocarcinomas, deregulation or genetic modification of HIPPO pathway components is common (Wu et al., 2018). This deregulation promotes nuclear localization and transcription factors binding to induce the transcription of anti-apoptotic genes. The resulting antiapoptotic proteins induce the development of cancer stem cell-like phenotypes and metastatic dissemination. Drug have been designed for targeting downstream effectors of the HIPPO signalling pathway (such as downstream transcription coactivators; YAP1 and TEAD) are currently in clinical trials (Ajani et al., 2021).

On the other hand, highly conserved Wnt signalling pathway is known to maintain adult tissue homeostasis and regulate embryonic development. Based on the observation genes associated with Wnt pathway are dysregulated in gastric adenocarcinomas, scientists have considered this pathway as a potential therapeutic target (Koushyar et al., 2020). Moreover, Notch 1/3 and Jagged 1/2 are upregulated in gastric adenocarcinoma, therefore, inhibiting their expression can be helpful in treating gastric adenocarcinoma. Although, preliminary evidence indicate that Hedgehog signalling pathways are deregulated in gastric adenocarcinomas (Koh et al., 2021); however, further investigations are required to confirm if targeting this pathway can be useful treatment option.

### Macrophages modulate therapeutic efficacy

The immune function and phenotypes of macrophages vary significantly depending on the cellular communication with immune microenvironments. This macrophage polarization plays contribute to inflammation and disease in the aspects of differentiation, survival, and instigation. Chronic inflammation triggers the production of pro-inflammatory transcription factors, while cancer cells attract macrophages by releasing cytokines and chemokines (TNF-α and IL-6) (Crusz and Balkwill, 2015; Chen et al., 2023). Macrophages cause cancer progression in mutagenic microenvironment by releasing inflammatory mediators (IL-6, TNF, and IFN-γ), growth factors like EGF and Wnt, reactive oxygen species, reactive nitrogen species, and proteases (Cassetta and Pollard, 2018). Tumor-associated macrophages (TAM)-derived IL-17 and IL-23 are associated with colon cancer development and progression, thus can play role in progression of gastric adenocarcinoma (Malesci et al., 2017).

Gastric cancer patients with high pretreatment TAM levels lived longer after receiving postoperative chemotherapy based on 5-fluorouracil (FU). Similarly, pretreatment high macrophage density was linked with an improved prognosis in patients treated with 5-FU adjuvant therapy (Malesci et al., 2017). Several immunotherapy approaches have been developed for cancer including tumor vaccines, adoptive cellular immunotherapy, antibodies, immune checkpoint inhibitors (ICIs), and small-molecule inhibitors. Several checkpoints connected with macrophages and T cells, which can be targeted for tumor growth inhibition (Weiskopf et al., 2016). Within the tumor microenvironment, reprogramming of macrophages regulates phagocytes and antigen-presenting cells. Macrophage phagocytosis if inhibited by inhibitors can suppress the immune system, thereby improving effectiveness of immunotherapy for gastric adenocarcinoma. Moreover, treatment based on targeting macrophages has been found more effective such as CSF-1R blockade with PLX3397 improved the efficacy of adoptive cell therapy against gastric cancer (Mok et al., 2014). The reported diversity and unique properties of TAMs in tumors can lead to create tailored therapeutic strategies, however, more in-depth research work is needed to unveil the role of TAMs and the underlying regulatory mechanisms associated with effective anti-tumor targets. Targeting TAMs can reverse tumor progression and play role in immune suppression, therefore targeting TAM may be potentially innovative therapeutic strategy in future (Chen et al., 2023).

### RNA N6-methyladenosine (m6A) modification

To overcome the complications associated with targeted therapy, chemotherapy and immunotherapy, it is important to identify diagnostic and prognostic markers, which can be used efficiently in gastric cancer (Brown et al., 2018; Moehler et al., 2022). In mammalian genes, one of the most abundant epitope transcriptome modifications is N6-methyladenosine, which is a methylation modification on the sixth N atom of adenine. This reversible modification results from the interaction among readers, writers, and erasers. Earlier studies have confirmed that N6-methyladenosine modification contribute to the progression of tumours as well as the treatment process (Du et al., 2022). We know that a series of improvements in immunotherapy, radiotherapy, chemotherapy, targeted therapy, photosensitive therapy, and minimally invasive surgery have increased the success rate of cancer treatment. Gastric adenocarcinoma treatment strategies should be improved because total mortality and drug resistance remain serious obstacles (Sun et al., 2023). In this regard m6A modification can be studied further as it has provided new hope for the treatment of different common cancers. In this regard, molecular docking-based techniques should be considered to determine immunotherapeutic targets. A potential target could also be the M6A modified RNA. Furthermore, m6A-related genes can serve not only as prognostic markers, but also as predictive markers of tumour occurrence, progression, and growth (Sun et al., 2023).

### JAK-STAT PATHWAY as a drug target in gastric adenocarcinoma

The Janus kinase (JAK) signal transducer and activator of transcription (JAK-STAT) pathway are involved in communication between cells and exterior environment (Xue et al., 2023). Activation of JAK-STAT signaling through cytokines, growth factors, interferons, and other specific molecules drive a series of physiological and pathological processes, including proliferation, metabolism, immune response, inflammation, and malignancy (Shitara et al., 2020; Xue et al., 2023). This JAK-STAT pathway can be targeted to develop effective therapeutic options for gastric adenocarcinoma such as cytokine or receptor antibodies, JAK inhibitors, and STAT inhibitors (Xue et al., 2023). Upstream cytokines and receptors are pivotal for modulating the functions of the JAK-STAT signaling pathway. Drugs responsible for manipulation of cytokines and receptors (such as tocilizumab that inhibits IL-6) can inhibit JAK-STAT, thus can be tested for the treatment of gastric adenocarcinoma. Most STAT inhibitors function by restricting STAT phosphorylation, inhibiting SH2-mediated dimerization, or inducing STAT degradation (Deodhar et al., 2022). Napabucasin enhances the efficacy of Bcl-2 inhibitor by blocking STAT3 and inducing DNA damage, thereby eliminating the immunosuppressive functions of the suppressor cells. Therefore, napabucasin has been recently approved by the FDA for the treatment of gastric cancer (Bitsch et al., 2022).

### Microsatellite instability in gastric cancer and the consequences for immune regulation and therapy

Mutations in the DNA mismatch repair (MMR genes), resulting from germline or somatic epi/genetic abnormalities, create the molecular phenotype called MSI, characterized by the accumulation of numerous mutations across the genome in the repetitive sequences (Velho et al., 2014). MSI occurs in approximately 15%–30% of gastric cancer cases (Pedrazzani et al., 2009). The MSI gastric cancer can occur sporadically, due to hereditary syndromes (e.g., Lynch syndrome), and exposure to *H. pylori* (Machado et al., 2009; Pedrazzani et al., 2009). The main mechanism by which MMR system failure occurs in MSI gastrointestinal cancers is genetic and epigenetic alterations at the MMR system effectors, namely, h-MLH1 and h-MSH2 (Peltomäki, 2001). In the progression of gastric carcinogenesis, members of the phosphatidylinositol 3-kinase (PI3K) and mitogen-activated protein kinase (MAPK) pathways are mutated and activated. Several studied specifically described mutations in the mixed lineage kinase 3 (MLK3), PIK3CA, KRAS, and epithelial growth factor receptor EGFR (Velho et al., 2010; Corso et al., 2011); Mutations in genes responsible for maintaining genomic integrity (e.g., MRE11, hMSH3, BLM, hMSH6, RAD50, MED1, and ATR) and cell cycle regulation and apoptosis (e.g., APAF1, BCL10, TGFβ, CASPASE5, RIZ, RII, TCF4, IGFIIR, BAX, and FAS) have been linked with MSI gastric cancer (Hudler, 2012). When MMR proteins are functionally lost, the phenotype is highly mutated with many missense and frameshift mutations, affecting key tumor suppressor genes and oncogenes. In contrast to microsatellite stable tumors (MSS), MSI cancers exhibit a 100- to 1000-fold increase in mutation rates (Hudler, 2012; Dudley et al., 2016). Microsatellites are particularly susceptible to replication errors; their repetitive sequences can be used to identify an intact or defective MMR (Hudler, 2012).

A major biomarker used to predict the benefit of ICIs across cancer types is MSI (Kavun et al., 2023). In MSI colorectal cancer, strong lymphocytic activation and the shift to a tumor microenvironment restrain metastatic potential, leading to a strong response to immunotherapy (Greco et al., 2023). A neoplastic cell with an MMR defect overexpresses several immune checkpoint proteins, including programmed death-ligand 1(PD-L1) and programmed death-1 (PD-1), that can be pharmacologically targeted, allowing the revival of cytotoxic immune response against the tumor (Greco et al., 2023).

Based on available data, gastric cancer patients need to have their MSI status evaluated for an accurate assessment of their prognosis. Gastric cancer is still considered a challenging cancer, despite all the recent advances in treatment. There is a need to gain a deeper understanding of the molecular aspects of MSI gastric cancer in order to develop new diagnostic and prognostic tools, as well as identify new therapeutic strategies and targets to treat gastric cancer (Velho et al., 2014).

## Conclusion

Gastric adenocarcinoma is one of the major healthcare burdens worldwide. It is needed to know the severity of symptoms and nature of progression for successful treatment options. For instance, endoscopic resection has gained high recognition and is known for improved outcomes in primary cancers or accidentally detected tumors. To develop novel personalized treatment options, it is necessary to understand the molecular biology and immunological regulations associated with gastric adenocarcinoma. Suitable postoperative treatment strategies for gastric adenocarcinoma can be personalized cancer vaccines, bispecific or trispecific antibodies, and antibody–drug conjugates. Considering the importance of immunological system, macrophages are crucial elements that can be considered or targeted for improved treatment outcomes. Investigating the detailed mechanisms based on the involvement of macrophages in progressions is important, as these macrophages are main triggering factors, and key regulators of immune system. Therefore, determining their unique properties in tumors can provide the groundwork for designing tailored therapeutic approaches. However, large-scale investigations are needed to unveil the role of essential molecules or signals in functional reprogramming of TAMs.

## Abbreviations

APC/MCC, Familial adenomatous polyposis; CIN, Chromosomal instability; CT, Computed tomography; DC, Destruction complex; EBV, Epstein-Barr virus; EMR, Endoscopic mucosal resection; ESD, Endoscopic submucosal dissection; EUS, Endoscopic ultrasonography; GS, Genomically stable; ICIs, Immune checkpoint inhibitors; JAK, Janus kinase; LEF, Lymphoid enhancer factor; LGR, Leucine-rich repeat-containing G-protein-coupled receptor; LRP, Lipoprotein receptor-related protein; MAPK, Mitogen-activated protein kinase; MLK3, Mixed lineage kinase 3; MMR, Mismatch repair; MSI, Microsatellite instability; PD-1, Programmed death-1; PD-L1, Programmed death-ligand 1; PET, Positron emission tomography; PI3K, phosphatidylinositol 3-kinase; RNF, Ring finger protein; TAM, Tumor-associated macrophages; TCF, T-cell factor; TCGA, The cancer genome atlas; TNM, Tumour-node-metastasis.
